# Functional characterization of nine critical genes encoding rate-limiting enzymes in the flavonoid biosynthesis of the medicinal herb *Grona styracifolia*

**DOI:** 10.1186/s12870-023-04290-z

**Published:** 2023-06-03

**Authors:** Chunzhu Xie, Ting Zhan, Jinqin Huang, Jun Lan, Lingling Shen, Hongbin Wang, Xiasheng Zheng

**Affiliations:** 1 Institute of Medicinal Plant Physiology and Ecology, School of Pharmaceutical Sciences, Guangzhou University of Chinese Medicine, Guangzhou, 510006 China; 2 The First Affiliated Hospital of Guangzhou University of Chinese Medicine, Guangzhou, 510405 China; 3 Guangzhou Analytical Applications Center, Shimadzu (China) Co., LTD, Guangzhou, 510010 China

**Keywords:** *Grona styracifolia*, Active flavonoids, Biosynthetic pathway

## Abstract

*Grona styracifolia* is a photophilous legume that contains abundant flavonoids with multiple pharmacological activities, which is used to cure urethral and biliary calculus in China for thousands of years. The authentication of the rate-limiting enzymes involved in the flavonoids biosynthesis pathway enabled a better understanding of the molecular aspect of quality formation and modulation of this medicinal herb. In this study, the chemical distribution characteristics and content of flavonoids in different tissues of *Grona styracifolia* were analyzed using ultraperormance liquid chromatography coupled with Q-TOF mass spectrometry and showed that active flavonoids were primarily synthesized and stored in the leaves. Subsequently, RNA sequencing (RNA-seq)-based transcriptome profiling of the different tissues revealed that the flavonoids biosynthesis in the leaves was the most active. Meanwhile, 27 full-length transcripts inferred encoding vital enzymes involved in the flavonoids biosynthesis were preliminarily excavated. Finally, four CHSs, four CHIs, and one FNSII were successfully characterized by heterologous expression, which involved in three rate-limiting steps of the flavonoid biosynthetic pathway. In conclusion, these results laid a foundation for further investigation of the molecular mechanism of the biosynthesis and modulation of active flavonoids in *Grona styracifolia*.

**Supplementary Information:**

The online version contains supplementary material available at 10.1186/s12870-023-04290-z.

## Background

*Grona styracifolia* (Osbeck) H. Ohashi & K. Ohashi is a semi-shrubby legume distributed across southern Asian countries, such as China, India, Thailand, et al. The dry aerial part of *Grona styracifolia* is widely used for curing urethral and biliary calculus in the clinic and thus derivates into several pattern medicines in China [1, 2]. Therefore, G*rona styracifolia* has been massively cultivated in southwestern China including Yunnan, Guangxi, and Guangdong, bringing about great economic value as well. *Grona styracifolia* produces abundant flavonoids, which possess multiple pharmacological activities. For example, schaftoside showed an activity of inhibiting the formation of cholesterol calculus [3], and isoorientin exhibited a protective effect on galactosamine-induced liver toxicity in mice [4]. Thus, the contents of active flavonoids within *Grona styracifolia* are considered important markers for evaluating the quality of this herb [1].

Flavonoids are a class of important secondary metabolites, which have a variety of biological functions in the process of plant growth, development, and adaptation to the environment, including ultraviolet defense, antioxidants, and attracting insects to pollinate and animals to disperse seeds [5, 6]. As a set of natural compounds, flavonoids possess a multitude of pharmacological activities in preventing cardiovascular and cerebrovascular diseases, cancer, obesity, and diabetes [7–10], and play a significant role in improving visual function, promoting anti-oxidation, anti-inflammatory and other aspects [11, 12]. Therefore, flavonoids have become a favor for scientists in the fields of drug research and development and healthcare product development.

**Fig. 1:**
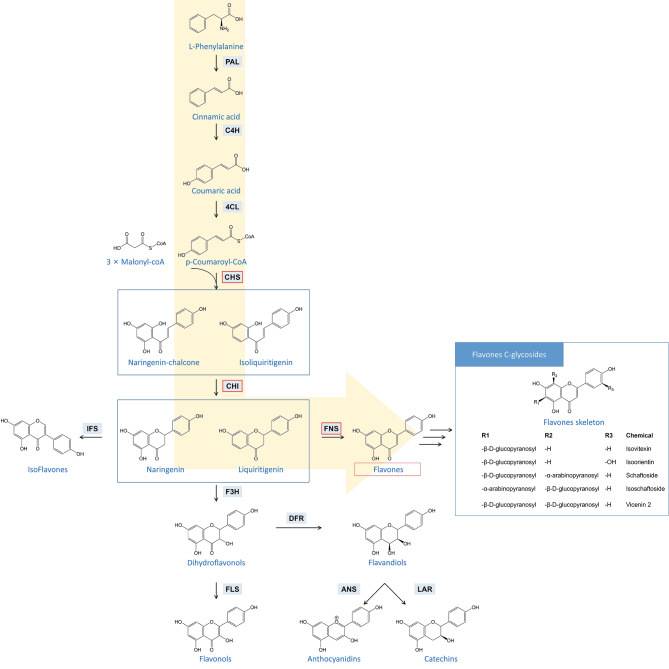
Predicted biosynthetic pathway of flavonoids in *Grona styracifolia*. The yellow arrow indicates the major pathway for flavones C-glycosides in *Grona styracifolia*

Naturally, the biosynthesis of flavonoids originates from the phenylpropanoid pathway described as followed [13, 14]. Phenylalanine is converted to 4-coumaroyl-CoA through a series of catalyzation reactions by phenylalanine ammonia-lyase (PAL), cinnamate-4-hydroxylase (C4H), and 4-coumaroyl-CoA ligase (4CL), respectively. Subsequently, a 4-coumaroyl-CoA coupled with three malonyl-CoA units to form chalcone through the catalyzation of chalcone synthase (CHS), which is the first rate-limiting enzyme during flavonoid biosynthesis. After that, a rapid cyclization reaction occurs under the catalysis of chalcone isomerase (CHI) and transforms chalcone into flavanone, which is the most important intermediate during the synthesis of flavonoids. Flavanone is positioned at a vital branch point of the flavonoids biosynthetic pathway, resulting in many variants on this basic skeleton, namely flavones, isoflavones, and dihydroflavonols, after catalyzed by flavone synthase (FNS), isoflavone synthase (IFS), and flavanone-3-hydroxylase (F3H), respectively. Finally, a series of decorations on the flavone skeleton under the catalyzation of UDP-glucuronosyltransferase (UGT) gives rise to a variety of glycosides, which had been previously isolated and identified from *Grona styracifolia* (see Fig. 1).

In this study, we combined transcriptomic and metabolomic approaches to investigate the tissue-specificity of the biosynthesis and accumulation of active flavonoids in *Grona styracifolia*. Subsequently, we further excavate potential elements involved in the biosynthetic pathway of flavonoids. Finally, those functional genes encoding rate-limiting enzymes in the biosynthesis of flavonoid parent nuclei were cloned and characterized. These results provided fundamental biosynthetic elements for future studies on the synthetic biology of active flavonoids in *Grona styracifolia*.

## Results

### Flavonoid metabolites profiling in ***Grona styracifolia***

**Fig. 2:**
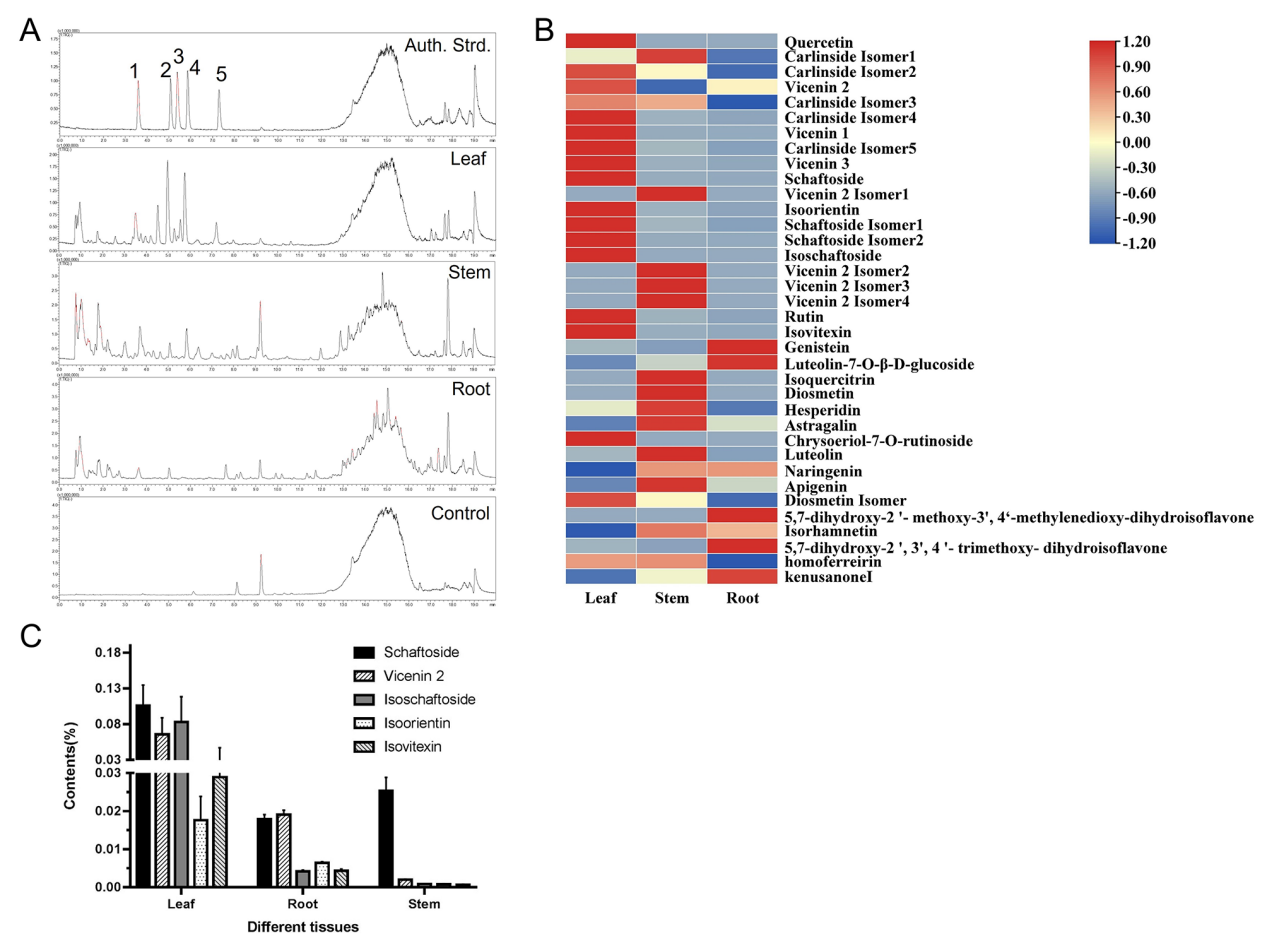
Chemical profiling of the flavonoids in different parts of *Grona styracifolia*. **(A)** Total ion chromatography (TIC) of extracts by UPLC-Q-TOF-MS/MS. **(B)** Heatmap of the relative contents of 36 identified flavonoids. **(C)** Distribution of five major active flavonoids in different tissues

Combined with ultraperformance liquid chromatography (UPLC) and Q-TOF-MS, we analyzed the flavonoid metabolites from the roots, stems, and leaves of *Grona styracifolia*, respectively (see Fig. 2A). As a result, a total of 36 flavonoid compounds were identified (Fig. 2B and Supplementary Table S1), with the quality control sample serving as a reference. In detail, the total relative content of flavonoids in the three tissues was leaf (128.5%) > stem (31.7%) > root (12.9%); while the distribution of flavonoid diversity behaves as stem (32) > leaf (27) > root (19). Furthermore, a quantitative and qualitative analysis against five representative active flavonoid glycosides, including vicenin 2, schaftoside, isoorientin, isoschaftoside, and isovitexin (see Fig. 2C and Supplementary Fig. S1), revealed that the contents of these compounds in the leaves were significantly higher than that in the roots and stems. To sum up, these results suggested that the most abundant content of flavonoids was observed in the leaves of *Grona styracifolia*, while the greatest diversity of flavonoids was found in its stems. This result uncovered the pharmacodynamic material basis of the aerial parts of *Grona styracifolia* for Chinese medicine treatment.

### Transcriptome analysis in different tissues and functional annotation

The above chemical profiling result revealed great differences in the contents and types of flavonoids in the roots, stems, and leaves of *Grona styracifolia*, which might be caused by the differences in gene transcription and regulation. Therefore, we sequenced the transcriptome of those three tissues, aiming to excavate functional genes involved in the flavonoid biosynthetic pathway and assess their expression pattern. A total of 114,695 unigenes were obtained out of 154,552 transcripts, with the N50 length of 1,395 bp and 1,770 bp, respectively (see Supplementary Table S2). We used the BUSCO pipeline to evaluate the assembly integrity, with a total of 75.6% complete orthologs identified, including 71.6% single-copy and 4.0% duplicates. At the same time, clean reads of each sample were mapped to the assembly and resulted in a mapped ratio of 78.93~86.95%, indicating the reasonable reliability of our assembly.

**Fig. 3:**
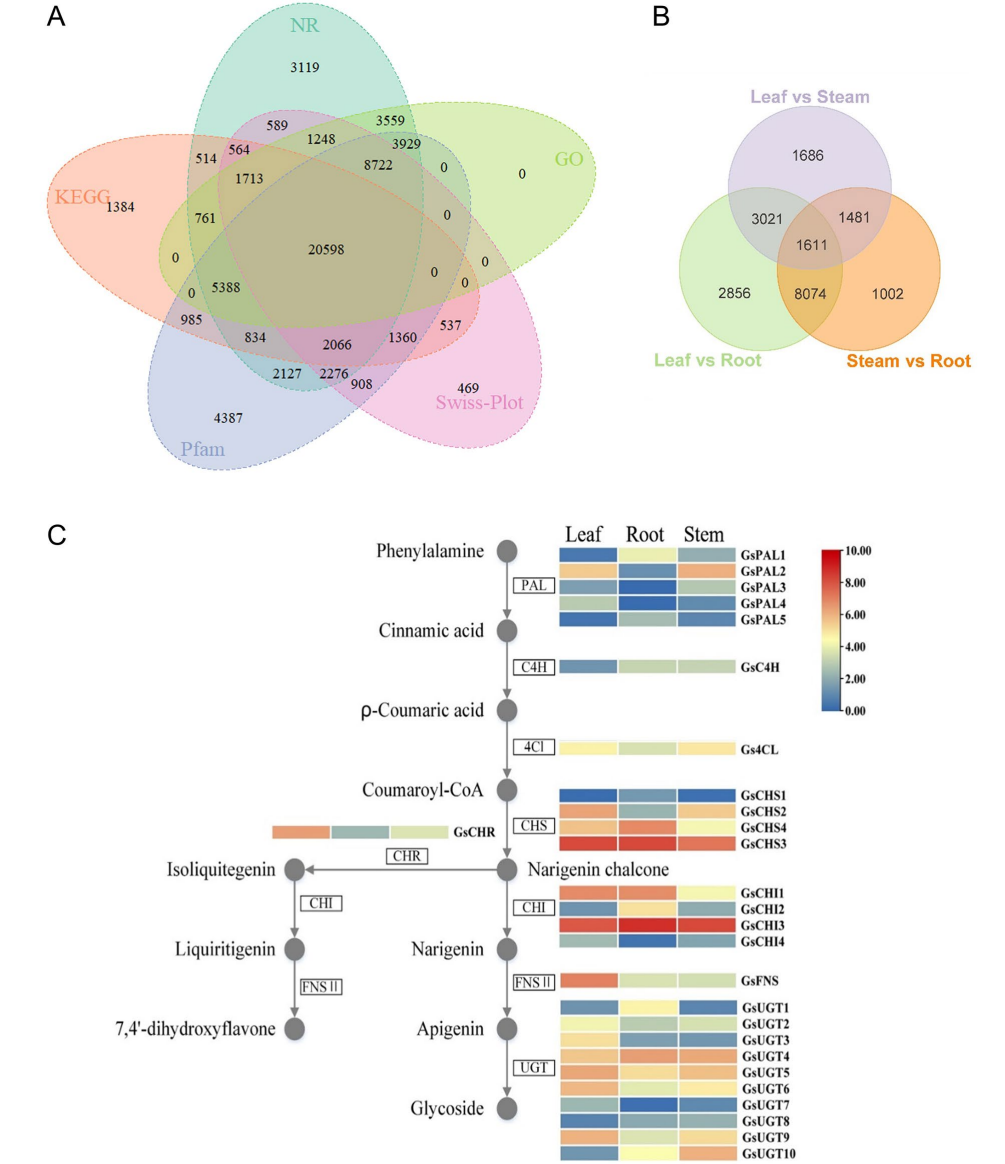
Functional annotation on the transcriptome of *Grona styracifolia*. **(A)** Venn diagram of annotation hit of those unigenes in our assembly against five public databases. **(B)** Differentially expressed genes (DEGs) in the pairwise comparison of the transcriptome data. **(C)** Predicted transcripts involved in the flavonoid biosynthetic pathway and their expression patterns

Altogether, 68,037 unigenes (59.32%) received functional annotation through five public databases, including GO, KEGG, NR, Swiss-Prot, and Pfam (see Fig. 3A), among which 69 transcripts were found to cover the entire biosynthetic pathway of flavonoids. At the same time, most of them were highly expressed in the leaves and stems in contrast to the roots. Besides, 19,731 transcripts were defined as differentially expressed genes (DEGs) (see Fig. 3B), among which 22 were involved in the biosynthetic pathway of flavonoids and were relatively active in the leaves. Using a combination of KEGG annotation, HMMER search, and local BLASTp, we found that twenty-seven transcripts were annotated as candidate genes involved in the flavonoid biosynthetic pathway (see Fig. 3C), coding for PAL (5), C4H (1), 4CL (1), CHS (4), CHI (4), FNS II (1), and UGT (10), respectively. From these results, we could conclude that the entire biosynthetic pathway of flavonoids was constantly and diffusely active in *Grona styracifolia*, resulting in plenty of flavonoids that might be essential to its physiological and/or resistant activities.

### Mining and functional characterization of the GsCHS genes

**Fig. 4:**
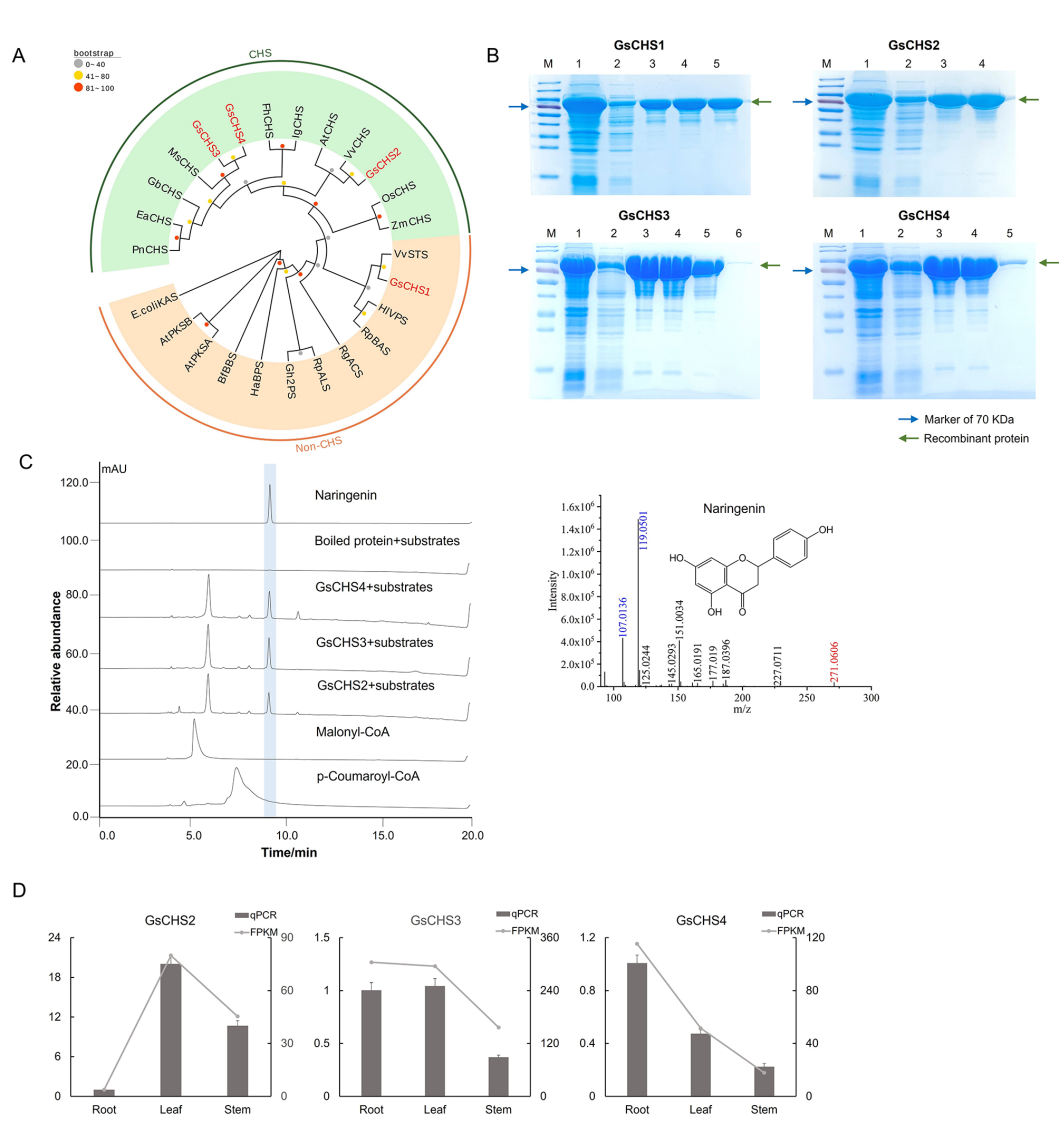
Excavation and functional characterization on CHS candidates from *Grona styracifolia*. **(A)** Phylogenetic tree of GsCHS candidates. **(B)** SDS-PAGE electrophoresis results for those GsCHS recombinant proteins, where lane 1 shows the total protein after induction, lane 2 shows the soluble protein, and lane 3 to 6 shows the purified recombinant proteins from the first to the fourth collected tube. **(C)** In vitro enzymatic activity assay result of GsCHS recombinant proteins and mass spectrum of the enzymatic products naringenin, with boiled protein and substrates served as the negative control. As in the mass spectrum, the number in red indicates the parental m/z and that in blue shows the major fragments. **(D)** Expression patterns of three GsCHS genes in the roots, stems, and leaves of *Grona styracifolia*

We identified four GsCHS gene candidates from the transcriptome data of *Grona styracifolia*, which all harbor two conserved domains (see Supplementary Fig. S2). The upstream one consists of four essential amino acid residues, including Cys164, Phe215, His303, and Asn336, which were defined as the catalytic machinery of the CHS protein. As for the downstream highly conserved motif *GFGPG*, it contains a large active site providing space for the tetrapeptide required for chalcone formation (i.e., naringenin and resveratrol) from one p-coumaroyl-CoA and three malonyl-CoA molecules. Then, a phylogenetic tree was constructed with the neighbor-joining (NJ) algorithm against these GsCHS candidates and a dozen well-characterized CHSs from other species (see Fig. 4A and Supplementary Table S3). The consequences displayed that GsCHS2, GsCHS3, and GsCHS4 were clustered with catalytic CHS, suggesting that they might encode functional redundant enzymes. Nonetheless, GsCHS1 was clustered with those type III polyketide synthases without CHS activities, indicating that it might lose its function. The results of this part provided a reasonable prediction for the subsequent functional characterization assays.

The open reading frames (ORFs) of those four GsCHS gene candidates were successfully amplified from the cDNA of *Grona styracifolia*. These ORFs were ligated into the prokaryotic expression vector pMAL-c5X and transferred into *E. coli* and induced by the IPTG stimulation, resulting in soluble recombinant proteins (see Fig. 4B, the full-length gels were presented in Supplementary Fig. S3). In vitro enzymatic reactions using the purified recombinant protein manifested that GsCHS2, GsCHS3, and GsCHS4 could catalyze the condensation reaction of one coumaroyl-CoA unit and three malonyl-CoA units into chalcone. Interestingly, the resulting chalcone, namely naringenin-chalcone in this study, is spontaneously isomerized into flavanone naringenin in vitro (see Fig. 4C). This phenomenon is consistent with a previous report [15]. Yet, the recombinant protein of GsCHS1 seemed to lose function, because no expected products were detected.

In addition, qRT-PCR was used to investigate the real expression patterns of the three catalytic CHSs in different tissues of *Grona styracifolia*. And the results suggested that the expression levels of *GsCHS2* and *GsCHS3* were the highest in the leaves, and *GsCHS4* was greatly expressed in the roots, followed by the leaves (see Fig. 4D). It was conjectured that the relatively high expression of GsCHS, a crucial functional gene in the flavonoid biosynthetic pathway, in the leaves contributes to a correspondence of the most vigorous biosynthesis and accumulation of flavonoids, which has been authenticated in the previous metabolic profiling.

### Prokaryotic expression and functional characterization of the GsCHI genes

**Fig. 5:**
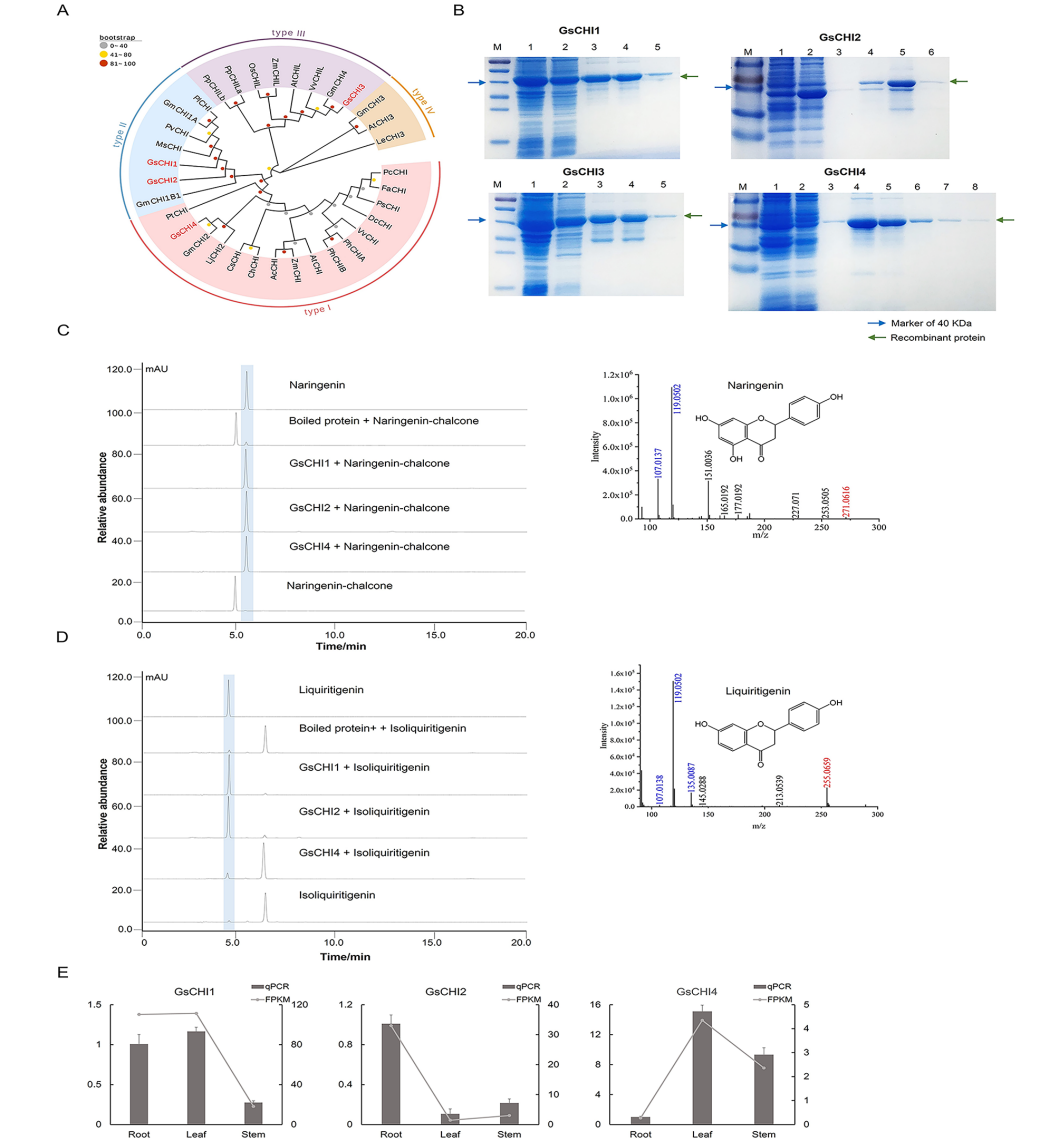
Excavation and functional characterization on CHI candidates from *Grona styracifolia*. **(A)** Phylogenetic tree of GsCHI candidates. **(B)** SDS-PAGE electrophoresis of recombinant GsCHI proteins, where lane 1 shows the total protein after induction, lane 2 shows the soluble protein, and lane 3 to 8 shows the purified recombinant proteins from the first to the sixth collected tube. **(C)** In vitro enzymatic activity assay result of GsCHI recombinant proteins and mass spectrum of the enzymatic products naringenin, with boiled protein and naringenin-chalcone served as the negative control. **(D)** In vitro enzymatic activity assay result of GsCHI recombinant proteins and mass spectrum of the enzymatic products liquiritigenin, with boiled protein and isoliquiritigenin served as the negative control. As in the mass spectrums, the number in red indicates the parental m/z and that in blue shows the major fragments. **(E)** Expression patterns of three GsCHI genes in the roots, stems, and leaves of *Grona styracifolia*

As mentioned above, four candidate GsCHI genes were screened from the transcriptome data of *Grona styracifolia*. Sequence alignment (see Supplementary Fig. S4) showed that except for GsCHI3, the other three *CHIs* all imbed typical essential amino acid loci for CHI proteins, including T50, Y108, and N115. An NJ tree was constructed with MEGA using these four GsCHI candidates and well-characterized ones from other species. And the phylogenetic results revealed (see Fig. 5A and Supplementary Table S4) that these GsCHIs fell into different clades. Among them, GsCHI1 and GsCHI2 were located in the evolutionary branch of type II CHI, suggesting that they might play a role in simultaneously catalyzing the conversion of naringenin-chalcone and isoliquiritigenin to naringenin and liquiritigenin, respectively. At the same time, GsCHI4 fell into the clade of type I CHI, indicating that it can only potentially catalyze the conversion of naringenin-chalcone to naringenin. In addition, GsCHI3 was positioned with type III CHIs, manifesting that it might lose its catalytic activity. Recombinant proteins of candidates CHI were eventually obtained (see Fig. 5B, the full-length gels were presented in Supplementary Fig. S5) and submitted to enzymatic reaction in vitro, with naringenin-chalcone and isoliquiritigenin serving as substrates. The results (Fig. 5C-D) were consistent with the above phylogenetic analysis and both type I and type II CHI coexist in leguminous plants as previously reported [16].

Finally, the expression patterns of the three functional *CHIs* in different tissues of *Grona styracifolia* were investigated, and the results demonstrated that (Fig. 5E) GsCHI1 was relatively active both in the leaves and roots, and the greatest expression of *GsCHI2* was found in the roots, while the most abundant expression of *GsCHI4* was in the leaves. These indicated that flavanone synthesis was more severe in the leaves and roots of *Grona styracifolia*. Combined with the metabolic data showing that relatively small amounts of flavanones were observed, it was speculated that the flavanones would immediately be converted into other products by various catalytic enzymes downstream of the biosynthetic pathway.

### Functional characterization of GsFNSII

**Fig. 6:**
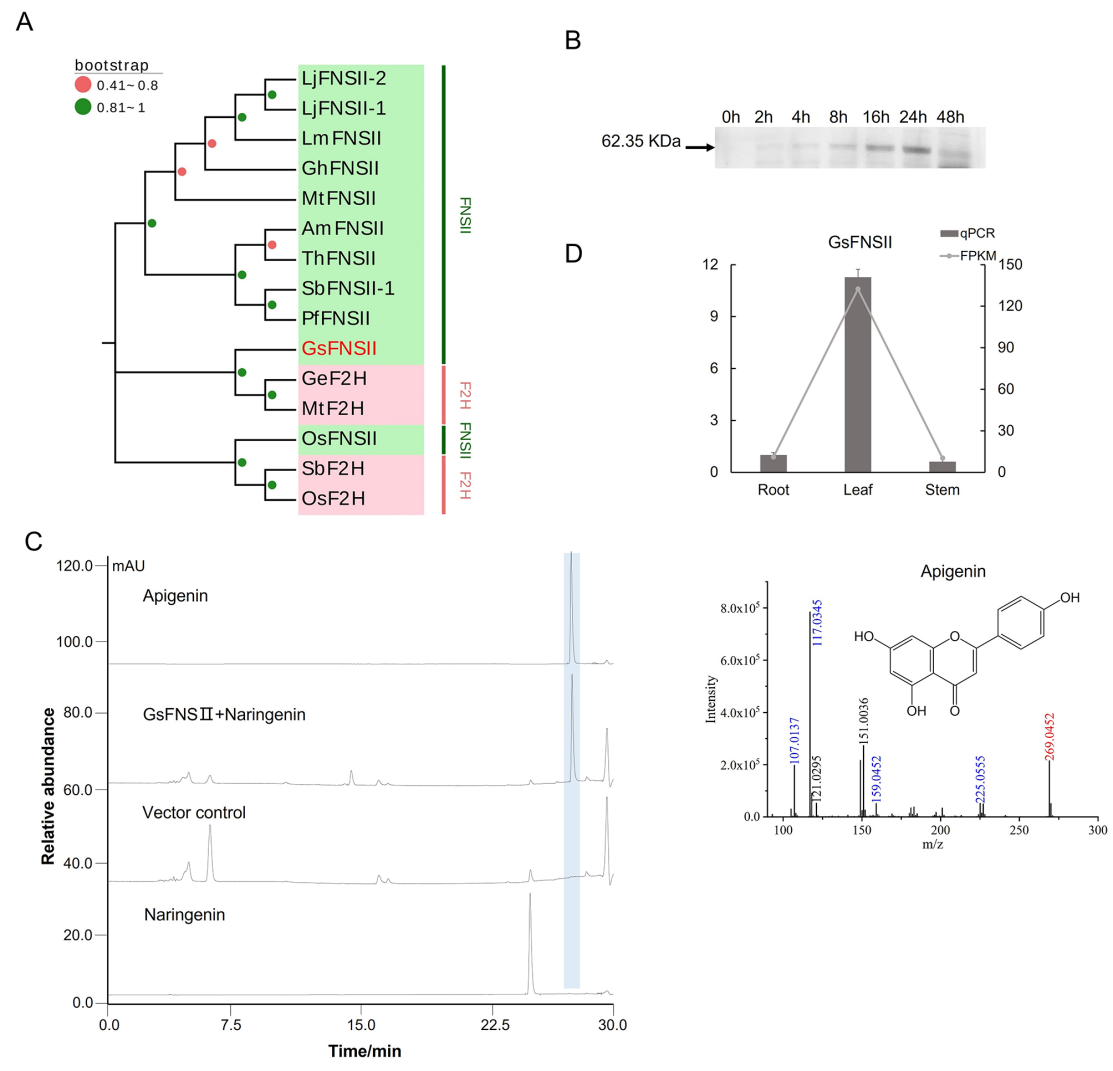
Excavation and functional characterization on FNSII candidates from *Grona styracifolia*. **(A)** Phylogenetic tree of GsFNSII candidates. **(B)** Protein expression of GsFNSII during 48 h of galactose induction. **(C)**In vivo enzymatic activity assay result of GsFNSII in *Saccharomyces cerevisiae* and mass spectrum of the enzymatic products apigenin, with yeast carrying the original vector served as the negative control. As in the mass spectrum, the number in red indicates the parental m/z and that in blue shows the major fragments. **(D)** Expression patterns of three GsFNSII genes in the roots, stems, and leaves of *Grona styracifolia*

In this study, only one candidate GsFNSII gene was excavated from the transcriptome data, which embeds four necessary and complete CYP450 conserved domains according to a multiple sequence alignment analysis (see Supplementary Fig. S6). Phylogenetic analysis between GsFNSII and multiple reported type II flavonoid synthase genes from other species showed that GsFNSII exhibited a close relationship with the flavonoid synthase in *Glycyrrhiza uralensis* and *Medicago sativa* within Leguminosae (see Fig. 6A and see Supplementary Table S5).

A recombinant pESC-URA yeast expression vector of GsFNSII was constructed, transferred into competent yeast strain WAT11, and induced by galactose. The expression of the recombinant protein was determined using western blotting to investigate the induction time. As a result, the recombinant protein of GsFNSII was detectable 2 h after induction and reached a peak amount at 24 h (see Fig. 6B, the full-length image for cropped gel was presented in Supplementary Fig. S7). Furthermore, the enzymatic function assay was conducted in *Saccharomyces cerevisiae* in vivo with naringenin used as the substrate. The result revealed that (Fig. 6C) GsFNSII functioned in catalyzing naringenin into apigenin in *Saccharomyces cerevisiae* WAT11. In addition, since no intermediate 2-hydroxyflavanone was detected in the enzymatic product, we speculated that the catalytic mechanism of GsFNSII was to directly convert flavanone into flavone. The expression patterns of GsFNSII in different tissues were investigated through qRT-PCR, which eventually demonstrated that (Fig. 6D) GsFNSII majorly expressed in the leaves, rather than in the roots and stems, indicating that the oxidative decoration took place in the leaves of *Grona styracifolia*.

## Discussion

Investigation of the accumulation of active components and elucidation on the biosynthesis pathway in medicinal plants benefits in improving our understanding of the mechanism underlying the quality construction and laying a foundation for future metabolic engineering. As a medicinal plant, *Grona styracifolia* produces abundant of bioactive flavonoid C-glycosides and exhibits stable and reliable pharmacological applications [17]. This study revealed that the biosynthesis of those active flavonoid C-glycosides were more active in the leaves rather than the stems and roots, indicating that the leaves are the target for following in-depth studies. In addition, it appears that such investigations are more feasible to perform on *Grona styracifolia*, compared to other medicinal plants like *Scutellaria baicalensis* and *Medicago truncatula*, where the biosynthesis and storage location of active ingredients are inconsistent [18, 19].

As a ubiquitous enzyme in higher plants, CHS belongs to the family of type III polyketide synthases. Studies have reported the coexistence of multiple CHS paralogous within one species [20, 21], yet, only one CHS plays a role in the biosynthesis of flavonoids mostly. In this study, four CHS candidate genes were obtained from *Grona styracifolia*, among which three are functional in vitro. This is consistent with that in *Clivia miniata* [22]. In addition, previous studies have demonstrated that CHS usually has a broad substrate preference and could catalyze both aromatic CoA and aliphatic CoA condensation reactions, i.e. the PpCHS from *Physcomitrella patens* [23] and the SbCHS from *Scutellaria baicalensis* [24]. Therefore, the substrate preference of the three GsCHSs needs to be further validated.

Although chalcones can be isomerized to flavanones spontaneously in plants, the reaction efficiency could be significantly improved under the mediation of CHI, making it an important element in the biosynthesis of flavonoids. Previous studies have suggested that type I chalcone isomerases can only catalyze the conversion of naringenin-chalcone to naringenin, which is common in vascular plants. However, both type I and type II CHI were found coexistent within legume plants [16]. For type III CHI, it loses the ability to catalyze the conversion of chalcone to flavanone due to the occupy of fatty acids in its catalytic domain. In this study, three catalytic CHIs, including one type I CHI and two type II CHIs, were identified from *Grona styracifolia*, while GsCHI3 has no catalytic activity in vitro. Combined with the results of sequence alignment and phylogenetic analysis, it was speculated that GsCHI3 might belong to type II CHI with no catalytic activity. In addition, enzymatic reaction results showed that there was functional duplication between GsCHI1 and GsCHI2, but they showed variable expression patterns in different tissues. It was speculated that there might be more precise functional differences between them, such as playing different roles in response to different biotic or abiotic stresses, which needs further exploration.

FNSII is a membrane-bound CYP450 enzyme, which relies on the assistance of NADPH and thus catalyzes the hydroxylation of substrates. Previous reports have shown that there are two types of catalytic mechanism for FNSII. For the first type, it catalyzes the substrate flavanone to form the intermediate 2-hydroxyflavanone and then converts it to the final product flavone, i.e. FNSII from *Antirrhinum majus* [25] and *Chrysanthemum indicum* [26]. For the other type, it directly converts flavanone to flavone without the formation of any intermediates, i.e. FNSII from soybean, *Gerbera jamesonii* [27], *Scutellaria baicalensis* [28], and *Perilla frutescens* [18]. In this study, no 2-hydroxyflavanone intermediate was detected in the yeast extract by LC-MS, suggesting that GsFNSII was to directly convert flavanone into flavone without producing the intermediate 2-hydroxyflavanone.

The three rate-limiting enzymes of flavonoid biosynthesis mentioned above, including CHS, CHI, and FNSII, exhibit in the form of duplicated genes in plants. The phenomenon of duplicated genes within a single species is mainly caused by DNA duplication either at the small-scale (tandem or segmental gene duplication), or at the large-scale (whole-genome duplication). To retain the duplication, their evolution strategy includes neofunctionalization and subfunctionalization, or otherwise pseudogenization under adaptive conflict [29]. Obviously, most CHS, CHI, and FNSII in *Grona styracifolia* survived as duplicated genes as they evolve in subfunctionalization. At the same time, several copies did not show expected enzyme activity in repeated experiments in vitro, which might be pseudogenes. Another possible explanation for this is that the microorganisms in our heterologous expression experiments can not provide the corresponding post-translational modification for those duplicated genes. Furthermore, it’s worth noting that the purpose of this study is to reveal the molecular basis of the quality formation of *Grona styracifolia*, by identifying those key elements of flavonoid biosynthesis. Those genes that did not show enzymatic activity were not further explored. In fact, it would be meaningful to study the functional divergence or functional redundancy for those duplicated genes in the future.

Due to the temporal and spatial limitations of the transcriptome, the biosynthetic elements obtained in this study reflect only those genes transcribed in vegetative organs of *Grona styracifolia* at the time of sampling, leaving a potential existence of other alternatives. A more systematic excavation would be flexible with the assistance of the whole genome sequence of this species.

Over the past decade, the rapid development of DNA sequencing technology has accelerated the study of functional genes in medicinal plants. Identification of key elements in the biosynthesis of important secondary metabolites lays a foundation for subsequent in-depth studies, such as transcriptional regulation and molecular breeding. As mentioned above, *Grona styracifolia* is sensitive to light and temperature, making it an ideal material for studying the effects of environmental factors on quality formation. Therefore, those biosynthetic elements authenticated in this paper pave a way for the study of the regulation of light and temperature in this plant in the future.

## Conclusions

In this study, we attempted to preliminarily investigate the biosynthesis and accumulation of active flavonoids in *Grona styracifolia*. Our result suggested that those active flavonoids were synthesized and restored majorly in the leaves. Furthermore, multiple vital elements involved in the biosynthetic pathway of flavonoids, including four CHSs, four CHIs, and one FNS, were successfully isolated and characterized. In conclusion, this study paved the way to a better understanding of the quality formation of flavonoid-rich medicinal herbs, thus providing a reference for future studies on elaborate metabolic modulation of medicinal plants.

## Materials and methods

### Plant material and metabolic extraction and detection

The *Grona styracifolia* material used in this study grew in the medicinal botanical garden at the Guangzhou University of Chinese Medicine. Fresh roots, stems, and leaves were sampled, cleaned with distilled water, placed in liquid nitrogen, and then stored in a refrigerator at -80 °C for subsequent processes.

Those three tissues of *Grona styracifolia* were grounded into fine powders with liquid nitrogen. One gram of each sample was accurately weighed, added with 10 mL of 80% methanol, and extracted with ultrasonic at 300 W for 30 min. The resulting extraction was then filtered with a 0.22 μm filter and submitted to liquid chromatographic assay on a Shimadzu SPD-M20 using a Shimadzu Shim-pack GIST C18-AQ HP column (2.1 mm × 100 mm, 1.9 μm), with gradient elution comprising solvent A (0.1% formic acid in water) and solvent B (methanol). The flow rate was set at 0.3 mL/min with the column temperature set as 40.0 °C. The MS conditions were set as followed: electrospray ionization (ESI), negative ion mode, interface voltage 3.0 kV, interface temperature 300 °C, atomizing gas flow 3.0 L/min, drying gas flow 10.0 L/min, and heating gas flow 10.0 L/min.

### RNA extraction and transcriptome sequencing

Total RNA was extracted from different tissues of *Grona styracifolia* using a plant total RNA purification kit (Tiangen, China) by following the manual. The integrity and quantity of the RNA samples were analyzed by 1% agarose gel electrophoresis and a NanoDrop 2000 C spectrophotometer (Thermo Scientific, USA). After that, quantified RNA samples were conducted to cDNA library construction, fragmented, ligated to adaptors, and purified to generate the sequencing libraries, which were then sequenced on an Illumina HiSeq 2000 platform. Paired-end reads with 150 bp were obtained and further examined through the software SeqPrep (https://github.com/jstjohn/SeqPrep) and Sickle (https://github.com/najoshi/sickle). Those resulting clean reads went through *de novo* assembly by Trinity ("https://github.com/trinityrnaseq/trinityrnaseq) to generate transcripts, among which the longest one within the same cluster was defined as the unigene. The software RSEM (http://deweylab.github.io/RSEM/) was used to quantify the expression level of those unigenes with FPKM (Fragments Per Kilobases per Million reads) as the index to gauge their relative expression levels.

### Candidate genes excavation

In addition to the common annotation against the KEGG database [30], we used a combination of BLAST and HMMER to mine candidate genes in the *Grona styracifolia* transcriptomic assembly as followed. Firstly, the software TransDecoder was used to predict open reading frames and possible peptide sequences with Uniprot annotation hit to form a potential protein sequence dataset against the transcriptomic assembly. Secondly, the amino acid sequences of the well-characterized reference gene were collected to form a query set and then used to run BLASTp against the above-predicted dataset with an e-value of 1e-5. Thirdly, the Hidden Markov Model (HMM) files of the conserved domain within corresponding reference genes from the Pfam database were used as queries to run the hmm-search software against the above-predicted dataset. Fourthly, resulting candidates from both BLASTp and hmm-search were merged and removed replicates, and then conducted to the CD-hit assay on the NCBI for further validation. Finally, those resulting candidate genes were screened manually regarding sequence identity, alignment length, and gene length.

### Phylogenetic analysis

The amino acid sequences of both candidate genes from *Grona styracifolia* and those of well-characterized ones from other species were downloaded and merged and then submitted to multiple sequence alignment using the Mega (V 7.0). After that, a model test was performed using Mega to assess the best model for each phylogeny assay. Finally, a phylogenetic tree was constructed using the neighbor-joining (NJ) algorithm with a bootstrap value set as 1,000. After the preliminary construction of the phylogenetic tree, the online tool Evolview (https://www.evolgenius.info/evolview/) was used to add detailed annotations to the phylogenetic tree.

### Prokaryotic expression and enzymatic reaction in vitro

The ORF of those target genes was cloned into the vector pEASY-blunt, which was then used to construct the prokaryotic expression vectors pMAL-c5X (for CHS candidates) and pET32a (for CHI candidates), and transformed into *E. coli* Rosetta (DE3). Subsequently, recombinant proteins were induced by the stimulation of IPTG, purified, and desalted. The resulting proteins were submitted to in vitro enzymatic reaction that was incubated at 30 ºC for 60 min. After that, 25 µL of 10% acetic acid solution was added to the system to terminate the reaction. Accordingly, 100 µL of ethyl acetate was added to the reaction system, vortexed for 5 min, and centrifuged at 12,000 rpm for 5 min, with the supernatant retained. The extraction by acetic acid was repeated one more time. Finally, two extracted solutions were combined, dried with nitrogen, redissolved with 200 µL of methanol, vortexed, and centrifuged at 16,000 rpm for 10 min, and the supernatant was submitted to HPLC-MS analysis.

### Eukaryotic expression and enzymatic reaction in vivo

The ORF of GsFNSII was inserted into the pEASY-blunt cloning vector, and then transferred into the eukaryotic expression vector pESC-Ura through homologous recombination and then transformed into the *Saccharomyces cerevisiae* strain WAT11. After induction by galactose for 2 h, the yeast was fed with 50 µL of naringenin (1 µg/µL) and incubated at 30 °C for 48 h to allow the enzymatic reaction in vivo. Then the yeast was collected and the metabolites in the cells were extracted by ultrasonic extraction for 30 min using methanol. Finally, the extract was filtered through a 0.22 μm microporous membrane, and then conducted to the HPLC-MS assay.

### HPLC-MS assay for enzymatic reaction products

Those enzymatic reaction products were analyzed on Agilent 1290 equipment with tandem detectors of a UV detector and a Q-TOF detector. We used an Agilent C_18_ column (4.6 mm × 150 mm, 2.5 μm) as the solid phase, and solvent A (water with 0.1% formic acid) and solvent B (methanol) as the mobile phase using gradient elution at a flow rate of 0.3 mL/min. For each sample, 2.0 µL extraction was injected into the HPLC-MS assay, with the column temperature set as 40.0 ºC. For the mass spectrum, we used the ESI as the ion source, and negative ion mode was selected with a full scan mode. The capillary voltage was set as 3,500 V, with the scan rate as 3 times per second. The gas flow rates were set as 11 L/min for the atomizer, and 8 L/min for drying, with a gas temperature of 300 ºC. The separator voltage acted as 65 V.

### qRT-PCR verification

Reverse transcription was performed against those RNA samples using an Evo M-MLV reverse transcription kit, in which potential genomic DNA was removed using DNase, and then the cDNA was synthesized. GsEF-1α was selected as the reference gene, and the cDNA was used as a template to determine the relative expression of target genes in different tissues. Three biological replicates were used with triplicates.

## Electronic supplementary material

Below is the link to the electronic supplementary material.

Supplementary Material 1

Supplementary Material 2

## Data Availability

The dataset supporting the conclusions of this article is available in the Global Pharmacopoeia Genome Database [31] (http://www.gpgenome.com/species/304). The raw sequencing data of RNA-seq has been deposited in the SRA of NCBI, with an accession number of PRJNA936623. The peptide sequences of those enzymes authenticated in this study were deposited in the NCBI as well, with accession numbers as followed: GsCHS1(OQ507796), GsCHS2(OQ507797), GsCHS3(OQ507798), GsCHS4(OQ507799), GsCHI1(OQ507800), GsCHI2(OQ507801), GsCHI3(OQ507802), GsCHI4(OQ507803), and GsFNSII(OQ507804).
