# Supplementary material for: Functional characterization of nine critical genes encoding rate-limiting enzymes in the flavonoid biosynthesis of the medicinal herb Grona styracifolia
Source: BMC Plant Biol. 2023 Jun 3;23:299. doi: 10.1186/s12870-023-04290-z (PMC10239141; doi:10.1186/s12870-023-04290-z)
Supplement: Supplementary file 1 — Supplementary Material 1 [file 12870_2023_4290_MOESM1_ESM.docx]

Supplementary Figures


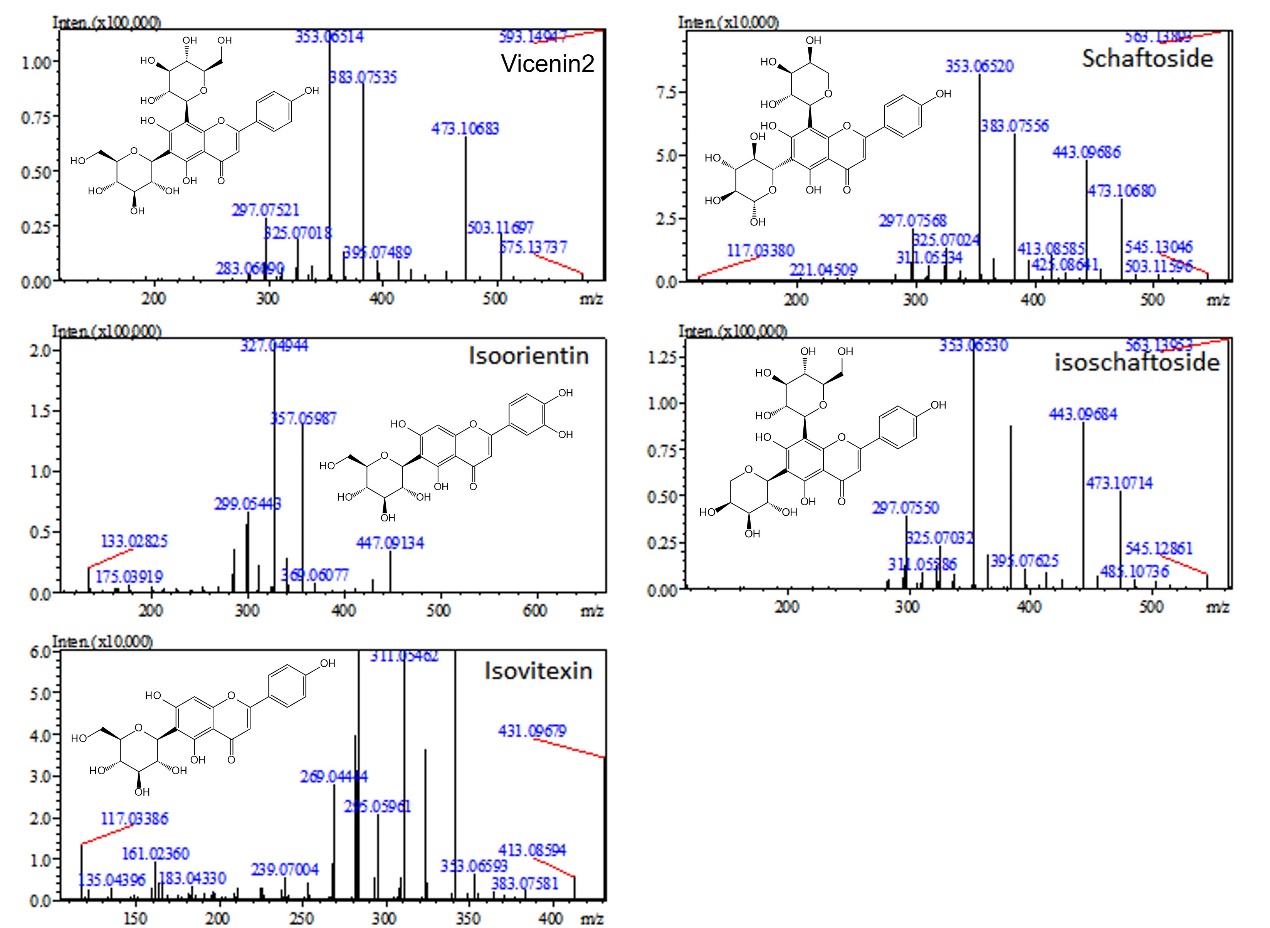


**Fig. S1.** Mass spectrum of the five major active flavonoids in *Grona styracifolia*


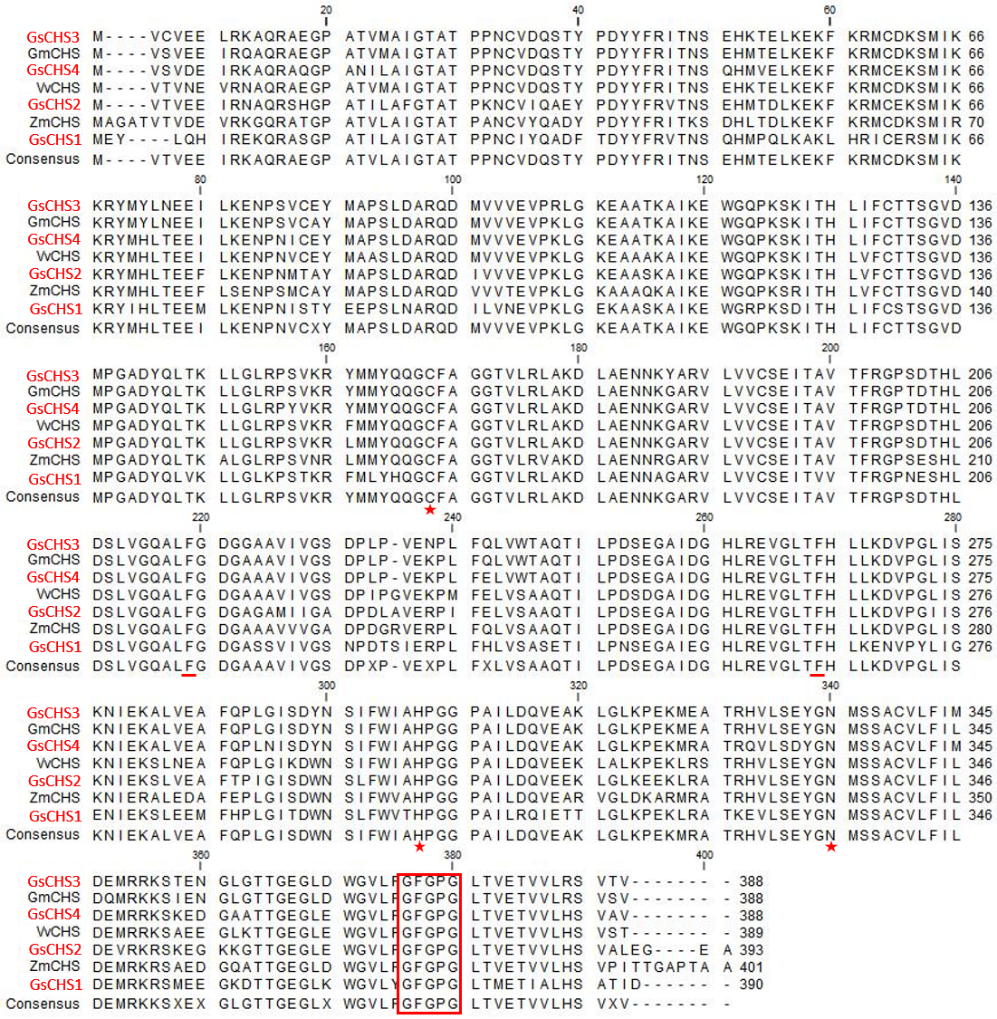


**Fig. S2.** Sequence alignment of four GsCHS candidates with several well characterized ones. The underscores indicate loci that determine substrate specificity. The asterisks indicate active sites, and boxes indicate conserved motifs.


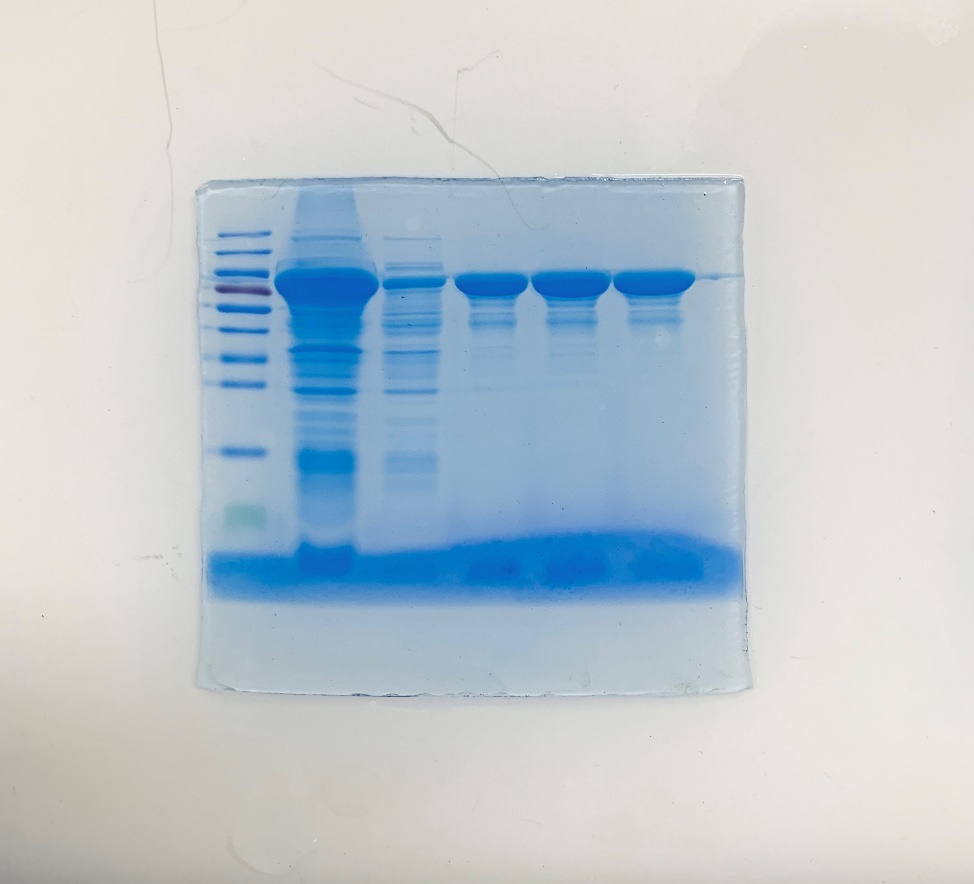

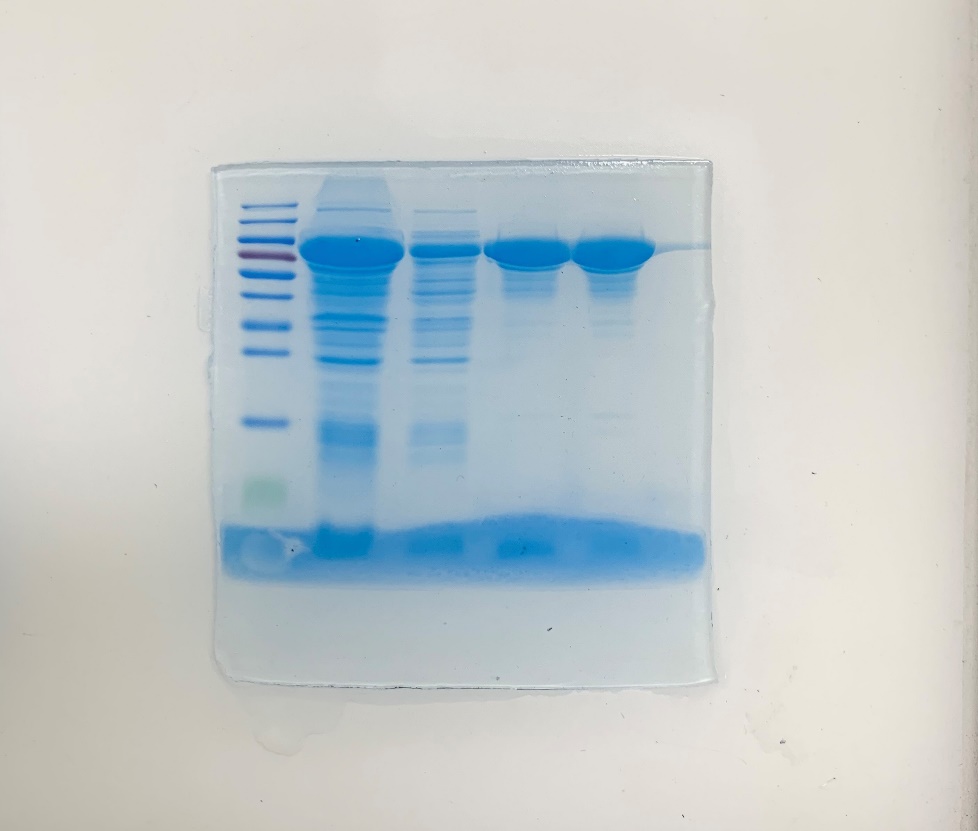


GsCHS1 GsCHS2


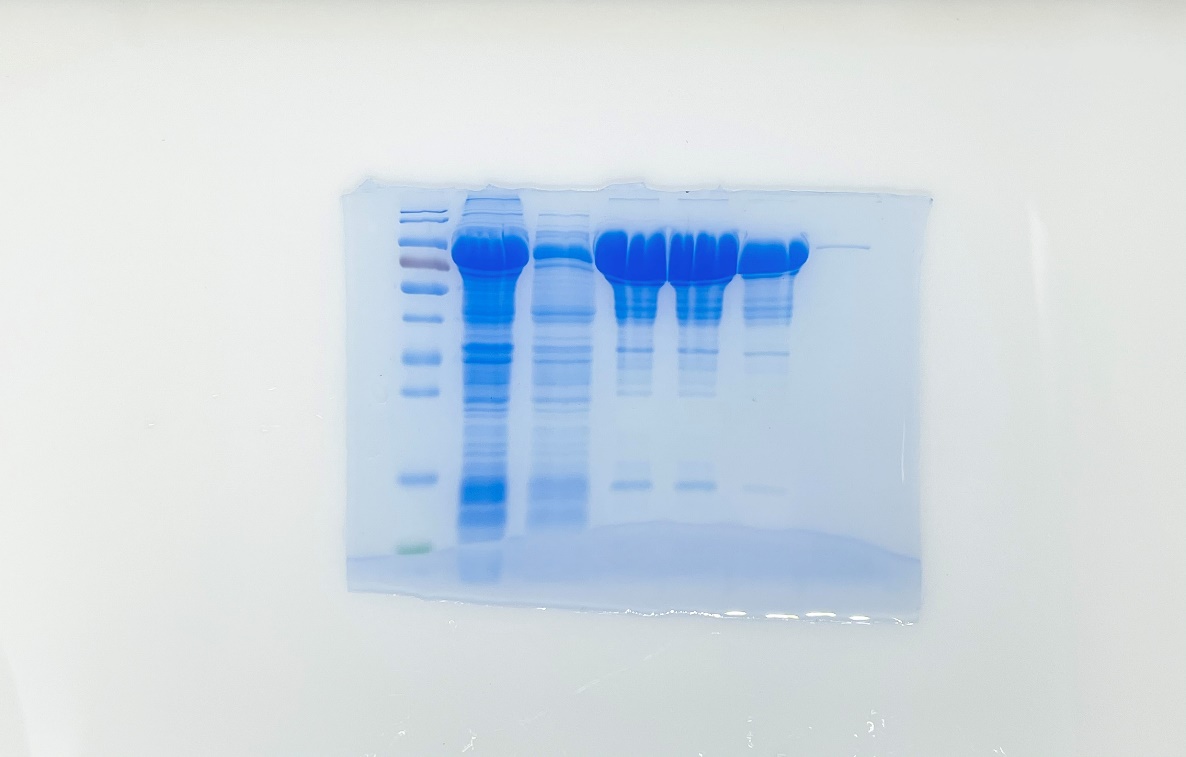

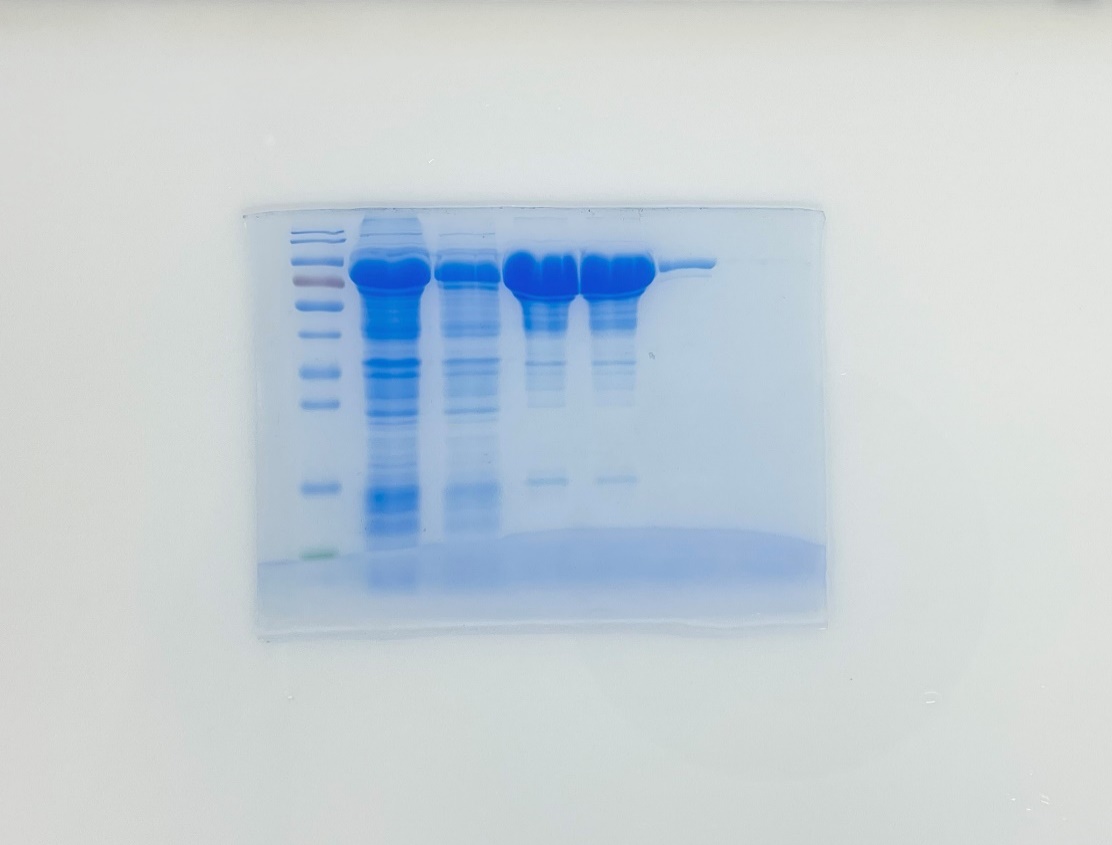


GsCHS3 GsCHS4

**Fig. S3.** Uncropped SDS-PAGE electrophoresis results for those GsCHS recombinant proteins.


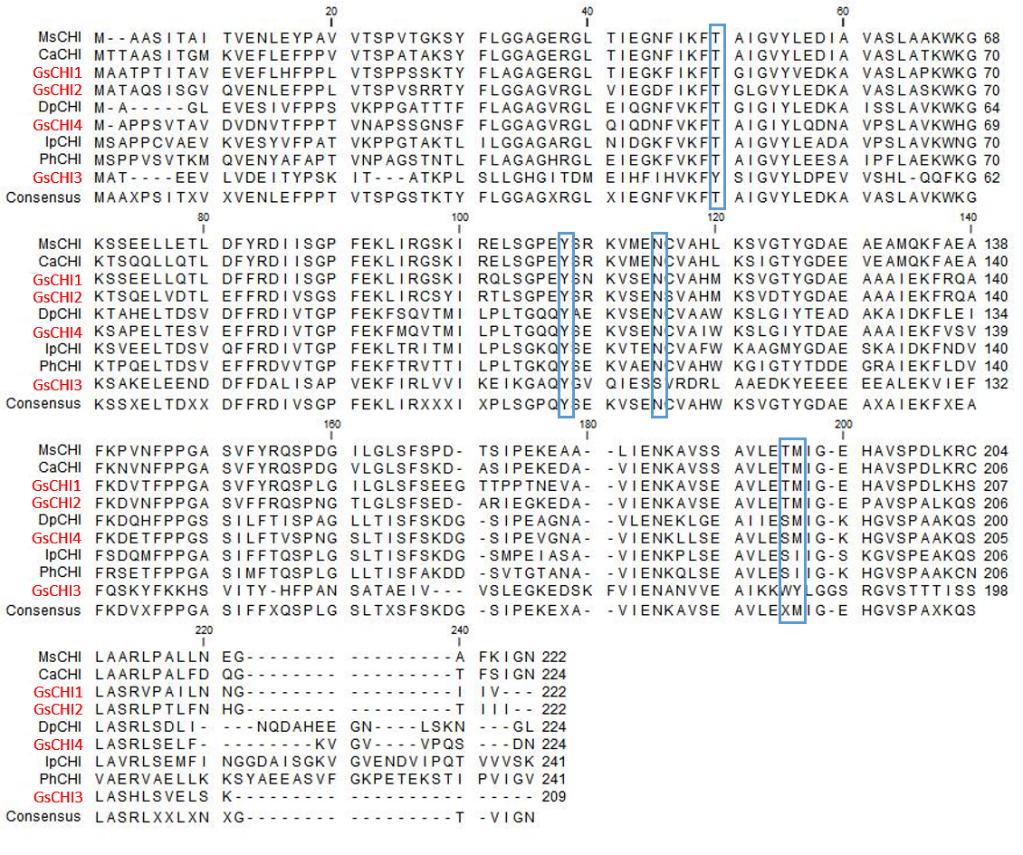


**Fig. S4.** Multiple sequence alignments on GsCHIs


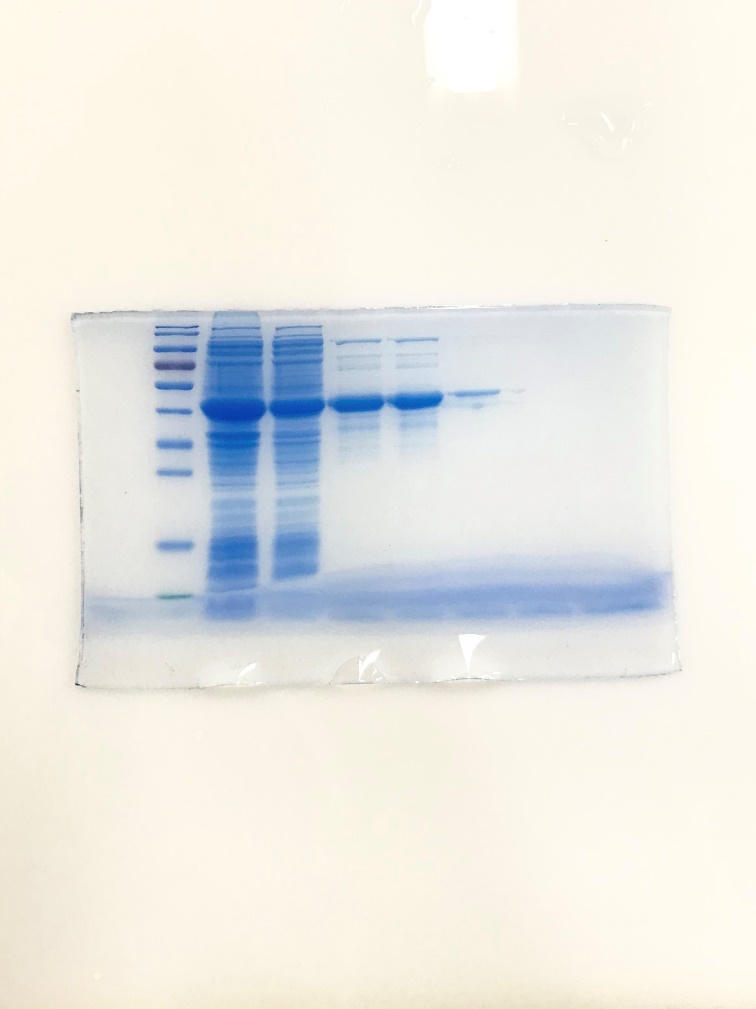

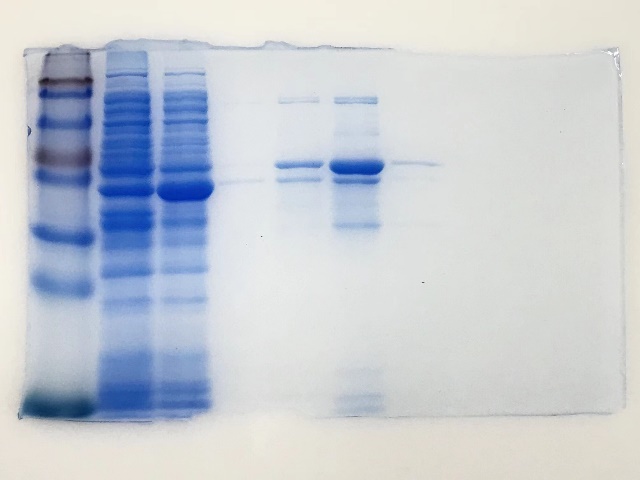


GsCHI1 GsCHI2


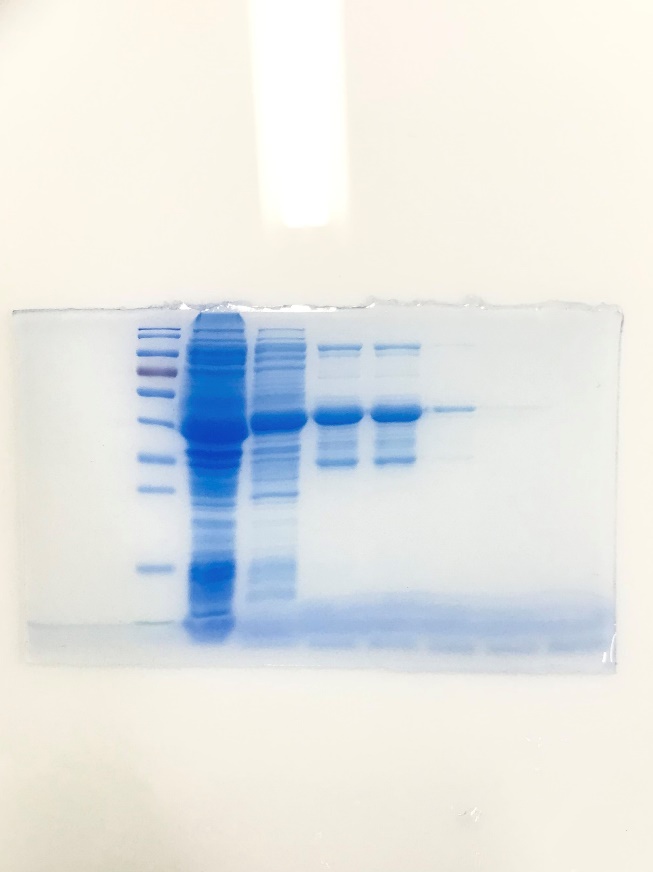

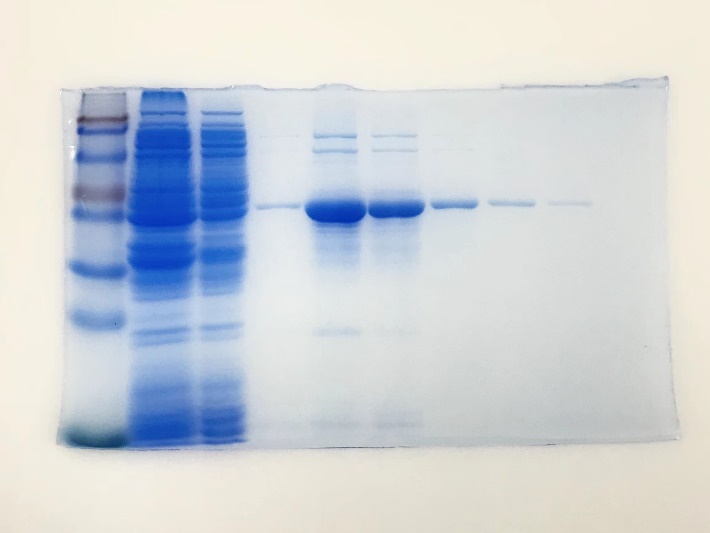


GsCHI3 GsCHI4

**Fig. S5.** Uncropped SDS-PAGE electrophoresis of recombinant GsCHI proteins


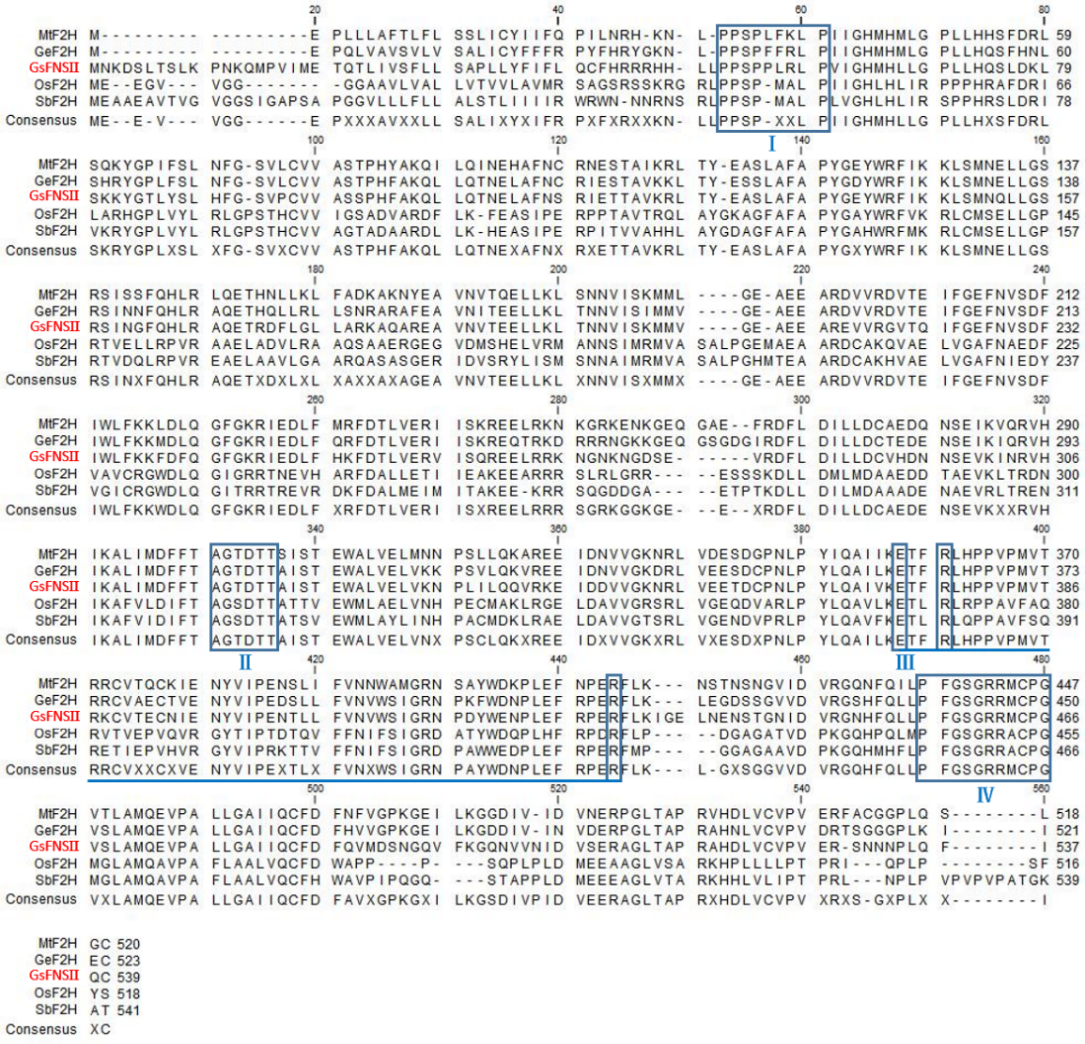


**Fig. S6.** Multiple sequence alignments on GsFNSⅡ


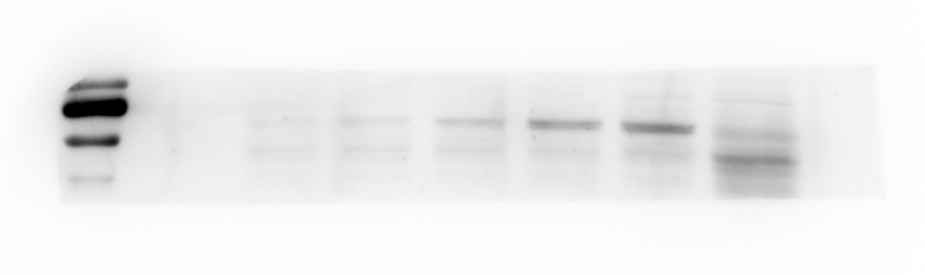


**Fig. S7.** Uncropped Western-blotting electrophoresis of the recombinant GsFNSⅡ protein
