# Supplementary material for: Functional characterization of nine critical genes encoding rate-limiting enzymes in the flavonoid biosynthesis of the medicinal herb Grona styracifolia
Source: BMC Plant Biol. 2023 Jun 3;23:299. doi: 10.1186/s12870-023-04290-z (PMC10239141; doi:10.1186/s12870-023-04290-z)
Supplement: Supplementary file 2 — Supplementary Material 2 [file 12870_2023_4290_MOESM2_ESM.doc]

Supplementary Tables

Table S1 Identified flavonoids in different tissues of *Grona styracifolia*

| N | Compound name | Retention time/min. | Formula | MS^1^/[M-H]^-^ | Error/ppm | Leaf (%Peak area) | Stem (%Peak area) | Roo t(%Peak area) |
| --- | --- | --- | --- | --- | --- | --- | --- | --- |
| 1 | Quercetin | 0.975 | C_15_H_14_O_9_ | 337.0569 | 1.276 | 0.600 | n.d. | n.d. |
| 2 | Carlinside Isomer | 2.864 | C_26_H_28_O_15_ | 579.1358 | 0.501 | 0.240 | 0.682 | n.d. |
| 3 | Carlinsid Isomer | 3.371 | C_26_H_28_O_15_ | 579.1337 | -3.194 | 0.270 | 0.136 | n.d. |
| 4 | Vicenin 2 | 3.501 | C_27_H_30_O_15_ | 593.1512 | -2.411 | 25.090 | 0.062 | 5.315 |
| 5 | Carlinside Isomer | 3.755 | C_26_H_28_O_15_ | 579.1335 | -3.505 | 0.260 | 0.225 | n.d. |
| 6 | Carlinside Isomer | 3.893 | C_26_H_28_O_15_ | 579.1339 | -2.884 | 1.930 | 0.051 | n.d. |
| 7 | Vicenin 1 | 3.946 | C_26_H_28_O_14_ | 563.1387 | -3.516 | 1.710 | 0.017 | 0.009 |
| 8 | Carlinside Isomer | 4.201 | C_26_H_28_O_15_ | 579.134 | -2.59 | 8.640 | 0.222 | n.d. |
| 9 | Vicenin 3 | 4.537 | C_26_H_28_O_14_ | 563.1391 | -2.344 | 3.300 | 0.060 | 0.009 |
| 10 | Schaftoside | 4.983 | C_26_H_28_O_14_ | 563.1409 | 0.408 | 37.320 | 0.418 | 0.351 |
| 11 | Vicenin 2 Isomer | 5.27 | C_27_H_30_O_15_ | 593.14883 | -3.979 | n.d. | 0.055 | n.d. |
| 12 | Isoorientin | 5.292 | C_21_H_20_O_11_ | 447.0917 | -3.601 | 1.400 | 0.159 | 0.118 |
| 13 | Schaftoside Isomer | 5.436 | C_26_H_28_O_14_ | 563.1391 | -2.717 | 0.160 | 0.020 | 0.008 |
| 14 | Schaftoside Isomer | 5.56 | C_26_H_28_O_14_ | 563.1392 | -2.539 | 0.390 | 0.012 | 0.009 |
| 15 | Isoschaftoside | 5.762 | C_26_H_28_O_14_ | 563.1401 | -0.941 | 41.580 | 0.034 | 0.028 |
| 16 | Vicenin 2 Isomer | 5.82 | C_27_H_30_O_15_ | 593.14892 | -3.827 | n.d. | 1.484 | n.d. |
| 17 | Vicenin 2 Isomer | 6.3 | C_27_H_30_O_15_ | 593.14971 | -2.495 | n.d. | 0.079 | n.d. |
| 18 | Vicenin 2 Isomer | 6.426 | C_27_H_30_O_15_ | 593.1495 | -2.849 | n.d. | 0.175 | n.d. |
| 19 | Rutin | 6.954 | C_27_H_30_O_16_ | 609.1456 | -0.804 | 1.530 | 0.052 | n.d. |
| 20 | Isovitexin | 7.211 | C_21_H_20_O_10_ | 431.09693 | -3.34 | 1.530 | 0.039 | 0.022 |
| 21 | Genistein | 7.631 | C_15_H_10_O_5_ | 269.0454 | -0.632 | 0.150 | 0.040 | 1.861 |
| 22 | Luteolin-7-O-β-D-glucoside | 7.721 | C_21_H_20_O_11_ | 447.0928 | -1.074 | 0.070 | 0.296 | 1.247 |
| 23 | Isoquercitrin | 7.865 | C_21_H_20_O_12_ | 463.08739 | -1.749 | n.d. | 0.030 | n.d. |
| 24 | Diosmetin | 8.18 | C_16_H_12_O_6_ | 299.0551 | -3.377 | n.d. | 20.971 | n.d. |
| 25 | Hesperidin | 9.14 | C_28_H_34_O_15_ | 609.182 | -0.755 | 0.020 | 0.052 | n.d. |
| 26 | Astragalin | 9.218 | C_21_H_20_O_11_ | 447.0921 | -2.773 | 0.180 | 2.963 | 0.756 |
| 27 | Chrysoeriol-7-O-rutinoside | 9.346 | C_28_H_32_O_15_ | 607.1658 | -0.708 | 0.190 | n.d. | n.d. |
| 28 | Luteolin | 11.989 | C_15_H_10_O_6_ | 285.0403 | -0.456 | 0.050 | 0.620 | 0.008 |
| 29 | Naringenin | 12.956 | C_15_H_12_O_5_ | 271.06125 | 0.184 | n.d. | 0.102 | 0.102 |
| 30 | Apigenin | 13.117 | C_15_H_10_O_5_ | 269.045 | -1.97 | 0.070 | 0.471 | 0.168 |
| 31 | Diosmetin Isomer | 13.325 | C_16_H_12_O_6_ | 299.0559 | -0.836 | 0.900 | 0.408 | n.d. |
| 32 | 5,7-dihydroxy-2 '- methoxy-3', 4‘-methylenedioxy-dihydroisoflavone | 13.395 | C_17_H_14_O_7_ | 329.06555 | -3.434 | n.d. | n.d. | 0.119 |
| 33 | Isorhamnetin | 13.8602 | C_16_H_12_O_7_ | 315.05095 | -0.254 | n.d. | 0.079 | 0.064 |
| 34 | 5,7-dihydroxy-2 ', 3', 4 '- trimethoxy- dihydroisoflavone | 14.428 | C_18_H_18_O_7_ | 345.0976 | -1.043 | 0.010 | n.d. | 0.156 |
| 35 | homoferreirin | 14.561 | C_17_H_16_O_6_ | 315.087 | -1.174 | 0.820 | 0.869 | n.d. |
| 36 | kenusanoneⅠ | 15.22 | C_21_H_22_O_6_ | 369.1331 | -3.522 | 0.080 | 0.829 | 2.535 |

Table S2 Statistics of the *de novo* assembly of the transcriptome sequencing.

| Type | Unigene | Transcript |
| --- | --- | --- |
| Total number | 114,695 | 15,4552 |
| Total base | 89,214,756 | 150,551,186 |
| Largest length (bp) | 14,768 | 14,768 |
| Smallest length (bp) | 201 | 201 |
| Average length (bp) | 777.84 | 974.11 |
| N50 length (bp) | 1,395 | 1,770 |
| Mean mapped percent (%) | 70.03 | 84.97 |
| GC percent (%) | 42.94 | 41.7 |

Table S3 Accession number and original species of those CHS proteins used in the phylogenetic analysis of this study.

| CHS | Accession Number | Species |
| --- | --- | --- |
| AtCHS | AAA32771.1 | *Arabidopsis thaliana* |
| RpBAS | AAK82824.1 | *Rheum palmatum* |
| HaBPS | AAL79808.1 | *Hypericum androsaemum* |
| RpALS | AAS87170.1 | *Rheum palmatum* |
| GbCHS | AAT68477.1 | *Ginkgo biloba* |
| VvSTS | ABV82966.1 | *Vitis vinifera* |
| HlVPS | ACD69659.1 | *Humulus lupulus* |
| FhCHS | AEO45114.1 | *Freesia hybrid cultivar* |
| OsCHS | BAA19186.2 | *Oryza sativa* |
| VvCHS | BAA31259.1 | *Vitis vinifera* |
| E.coliKAS | BAA35899.2 | *Escherichia coli* |
| PnCHS | BAA87922.1 | *Psilotum nudum* |
| IgCHS | BAE53636.1 | *Iris germanica* |
| BfBBS | CAA10514.1 | *Bromheadia finlaysoniana* |
| ZmCHS | CAA42763.1 | *Zea mays* |
| RgACS | CAC14058.1 | *Ruta graveolens* |
| AtPKSA | O23674.1 | *Arabidopsis thaliana* |
| MsCHS | P30074.1 | *Medicago sativa* |
| Gh2PS | P48391.2 | *Gerbera hybrid cultivar* |
| AtPKSB | Q8LDM2.1 | *Arabidopsis thaliana* |
| EaCHS | Q9MBB1.1 | *Equisetum arvense* |

Table S4 Accession number and original species of those CHI proteins used in the phylogenetic analysis of this study.

| CHI | Accession Number | Species |
| --- | --- | --- |
| PcCHI | A5HBK6.1 | *Pyrus communis* |
| PhCHIA | AAF60296.1 | *Petunia x hybrida* |
| AtCHI3 | AAM61303.1 | *Arabidopsis thaliana* |
| LeCHI3 | AAQ55182.1 | *Solanum lycopersicum* |
| GmCHI1A | AAT94358.1 | *Glycine max* |
| GmCHI1B1 | AAT94359.1 | *Glycine max* |
| GmCHI2 | AAT94360.1 | *Glycine max* |
| GmCHI3 | AAT94361.1 | *Glycine max* |
| GmCHI4 | AAT94362.1 | *Glycine max* |
| AcCHI | AAU11843.1 | *Allium cepa* |
| ChCHI | ABM64798.1 | *Gossypium hirsutum* |
| PsCHI | ADK55061.1 | *Paeonia suffruticosa* |
| CsCHI | BAA36552.1 | *Citrus sinensis* |
| PhCHIB | CAA32730.1 | *Petunia x hybrida* |
| ZmCHI | CAA80441.1 | *Zea mays* |
| LjCHI | CAD69022.1 | *Lotus japonicus* |
| AtCHIL | NP_568154.1 | *Arabidopsis thaliana* |
| OsCHIL | NP_001065587.1 | *Oryza sativa* |
| ZmCHIL | NP_001151452.1 | *Zea mays* |
| PcCHI | A5HBK6.1 | *Pyrus communis* |
| PvCHI | P14298.2 | *Phaseolus vulgaris* |
| MsCHI | P28012.1 | *Medicago sativa* |
| AtCHI | P41088.2 | *Arabidopsis thaliana* |
| VvCHI | P51117.1 | *Vitis vinifera* |
| FaCHI | Q4AE11.1 | *Fragaria x ananassa* |
| LjCHI2 | Q8H0G1.1 | *Lotus japonicus* |
| PlCHI | Q43056.1 | *Pueraria montana* |
| DcCHI | Q43754.1 | *Dianthus caryophyllus* |
| PpCHILb | XP_001769093.1 | *Physcomitrella patens* |
| PpCHILa | XP_001773128.1 | *Physcomitrella patens* |
| VvCHIL | XP_002280158.1 | *Vitis vinifera* |
| PtCHI | XP_002315258.1 | *Populus trichocarpa* |

Table S5 Accession number and original species of those FNSⅡ proteins used in the phylogenetic analysis of this study.

| FNSⅡ | Accession Number | Source |
| --- | --- | --- |
| GhFNSⅡ | AAD39549.1 | *Gerbera hybrid cultivar* |
| MtFNSⅡ | ABC59104.2 | *Medicago truncatula* |
| MtF2H | ABC86159.1 | *Medicago truncatula* |
| LjFNSⅡ-1 | AMQ91109.1 | *Lonicera japonica* |
| LjFNSⅡ-2 | AMQ91111.1 | *Lonicera japonica* |
| LmFNSⅡ | AMQ91113.1 | *Lonicera macranthoides* |
| SbFNSⅡ-1 | AMW91728.1 | *Scutellaria baicalensis* |
| GeF2H | BAA22423.1 | *Glycyrrhiza echinata* |
| AmFNSⅡ | BAA84071.1 | *Antirrhinum majus* |
| ThFNSⅡ | BAA84072.1 | *Torenia hybrid cultivar* |
| PfFNSⅡ | BAB59004.1 | *Perilla frutescens* |
| OsF2H | BAG94143.1 | *Oryza sativa* |
| OsFNSⅡ | BAG94859.1 | *Oryza sativa* |
| SbF2H | XP_002461286.1 | *Sorghum bicolor* |
